# Maintenance therapy in acute myeloid leukemia after allogeneic hematopoietic stem cell transplantation

**DOI:** 10.1186/s13045-020-01017-7

**Published:** 2021-01-06

**Authors:** Li Xuan, Qifa Liu

**Affiliations:** Department of Hematology, Nanfang Hospital, Southern Medical University, Guangzhou, 510515 China

**Keywords:** Maintenance therapy, Acute myeloid leukemia, Targeted drugs, Allogeneic hematopoietic stem cell transplantation

## Abstract

Relapse remains the main cause of treatment failure in acute myeloid leukemia (AML) undergoing allogeneic hematopoietic stem cell transplantation (allo-HSCT). Emerging evidence has demonstrated that AML patients might benefit from maintenance therapy post-transplantation, especially for high-risk AML patients. In this mini-review, we will summarize targeted drugs, such as hypomethylating agents, FLT3 inhibitors and isocitrate dehydrogenase inhibitors, as maintenance therapy post-transplantation in AML patients undergoing allo-HSCT.

## Background

Acute myeloid leukemia (AML) is a heterogeneous group of clonal diseases. Conventional chemotherapy can result in the complete remission (CR) rate of approximately 70–80% in AML patients [1, 2]. Leukemia relapse remains the main cause of treatment failure, including the patients undergoing allogeneic hematopoietic stem cell transplantation (allo-HSCT) [3]. Therefore, there is an urgent need to identify an effective and safe approach to improve post-remission survival. Conventional chemotherapy as maintenance therapy might have no benefit to most AML patients, as the efficacy is often offset by treatment-related mortality [4, 5]. Recently, novel targeted drugs have been used as maintenance therapy in patients with AML, including maintenance after allo-HSCT [6–9]. Some studies have shown that AML patients might benefit from maintenance with targeted drugs post-transplantation [6–9]. In this study, we do a mini-review about targeted drugs as maintenance post-transplantation in AML patients undergoing allo-HSCT.

The broad definition of maintenance therapy includes preemptive therapy based on measurable residual disease (MRD) and prophylactic therapy not based on MRD. The narrow definition of maintenance therapy refers only to prophylactic therapy. It remains under discussion whether preemptive or prophylactic therapy is superior in reducing relapse and improving survival for AML patients [10, 11]. Here, we mainly focus on prophylactic therapy post-transplantation.

## Hypomethylating agents

Hypomethylating agents such as azacitidine and decitabine have shown favorable efficacy and tolerability as induction therapy in AML patients, especially elderly patients unable to tolerate intensive chemotherapy [12, 13]. Some retrospective and small-sample prospective studies demonstrated that maintenance therapy with hypomethylating agents post-transplantation was safe and could reduce relapse, thereby prolonging the survival of AML patients, especially high-risk AML patients [14–17]. On the contrary, other studies suggested that AML patients might not benefit from maintenance with hypomethylating agents after allo-HSCT [18, 19]. Recently, a prospective phase II trial revealed that preemptive therapy with azacitidine could prevent or delay hematological relapse in patients with high-risk AML or myelodysplastic syndrome (MDS) who achieved CR after chemotherapy or allo-HSCT [20]. Another prospective phase I/II study revealed that CC-486, an oral formulation of azacitidine, was well tolerated as maintenance post-transplantation in patients with AML or MDS undergoing allo-HSCT, with the 1-year relapse rate of 21% [21]. It was a pity that both studies were single-armed. A phase II randomized controlled trial (RCT) from China has demonstrated that minimal-dose decitabine maintenance combined with recombinant human granulocyte colony-stimulating factor after allo-HSCT could reduce relapse for high-risk AML patients undergoing allo-HSCT, with the 2-year relapse rate of 15.0% and 38.3% in the intervention and non-intervention groups [22]. Nowadays, there are few reports comparing the effect of preemptive or prophylactic use with hypomethylating agents post-transplantation on the outcomes of AML patients undergoing allo-HSCT. Key studies of hypomethylating agents as maintenance strategy in AML patients after allo-HSCT are summarized in Table 1.

**Table 1:** Key studies of hypomethylating agents as maintenance therapy in AML patients after allo-HSCT

References	Study design	Patients, N	Maintenance therapy regimen	Relapse	Survival
Jabbour [14]	Retrospective, single arm	8 acute leukemia (7 AML, 1 ALL)	low-dose AZA daily × 5 days every 28 days for a median of 8 cycles	3/8 (37.5%)	5 alive with CR, 2 alive with leukemia
de Lima [15]	Phase I	45 high-risk AML/ MDS (37 AML, 8 MDS)	low-dose AZA daily × 5 days every 30 days for a maximum of 4 cycles	24/45 (53.3%)	1-year OS: 77%; 1-year LFS: 58%
Maples [18]	Retrospective, 2 arms	25 (18 AML, 7 MDS)	AZA 32 mg/m^2^/day × 5 days every 28 days for 4–6 cycles	16% (AZA) versus 14% (Control)	1-year OS: 60% (AZA) versus 64% (Control)
Oshikawa [19]	Retrospective, 2 arms	10 high-risk AML	AZA (30 mg/m^2^/day on days 1–7) and GO (3 mg/m^2^ on day 8) every 4 weeks for up to 4 cycles	AZA-GO: 4/10 (40.0%)	AZA-GO versus Control 1-year OS: 70.0% versus 59.8%; 1-year LFS: 60.0% versus 42.8%
Pusic [16]	Phase I	22 (17 AML, 5 MDS)	DAC × 5 days every 6 weeks for a maximum of 8 cycles	2-year CIR: 28%	2-year OS: 56%; 2-year LFS: 48%
Ma [17]	Retrospective, 2 arms	21 high-risk AML	DAC 20 mg/m^2^/day × 5 days every 3 months for up to 4–6 cycles	3-year CIR: 5.9% (DAC) versus 45.3% (Control)	3-year OS: 92.9% (DAC) versus 51.8% (Control)
Platzbecker [20]	Phase II	24 high-risk AML or MDS with MRD-positive post-transplantation	preemptive therapy with AZA 75 mg/m^2^/day on days 1–7 of a 29-day cycle for a minimum of 6 cycles	8/24 (33.3%)	2-year OS: 62%; 2-year LFS: 54%,
de Lima [21]	Phase I/II	30 (26 AML, 4 MDS)	CC-486 daily × 7 days every 28 days for up to 12 cycles	1-year CIR: 21%	1-year OS in the 7-day and 14-day dosing cohorts of 86% and 81%
Gao [22]	Phase II RCT	202 high-risk MRD- negative AML (G-DAC: 100; Non-G-DAC: 102)	G-DAC: 5 mg/m^2^ of DAC on days 1–5 and 100 mg/m^2^ of rhG-CSF on days 0–5 every 6–8 weeks for up to 6 cycles; Non-G-DAC: no intervention	2-year CIR: 15.0% (G-DAC) versus 38.3% (Non-G-DAC)	G-DAC versus Non-G-DAC 2-year OS: 85.8% versus 69.7%; 2-year LFS: 81.9% versus 60.7%

*Ref* reference, *N* number, *AML* acute myeloid leukemia, *ALL* acute lymphoblastic leukemia, *AZA* azacitidine, *CR* complete remission, *MDS* myelodysplastic syndrome, *OS* overall survival, *LFS *leukemia-free survival, *GO* gemtuzumab ozogamicin, *DAC* decitabine, *CIR* cumulative incidence of relapse, *MRD* measurable residual disease, *RCT* randomized controlled trial, *rhG-CSF* recombinant human granulocyte colony-stimulating factor

## FLT3 inhibitors

FLT3 internal tandem duplication (FLT3-ITD) mutations occur in approximately 25% of adults with AML. In contrast to AML with FLT3 wild-type, AML patients with FLT3-ITD mutations have shorter remissions and higher relapse rates [23, 24]. Allo-HSCT could improve the survival of patients with FLT3-ITD AML, but leukemia relapse remains high [23–25]. Currently, FLT3 inhibitors, including sorafenib, midostaurin, gilteritinib, quizartinib and crenolanib, have been applied to clinical practice [23, 24]. They have been explored in various settings for patients with FLT3-ITD AML, including induction, post-remission maintenance pre- and post-transplantation and salvage therapy for refractory relapsed patients [23–33]. Besides, FLT3 inhibitors have been also explored in the treatment of AML patients without FLT3 mutations [26, 34]. Growing evidence has suggested that patients with FLT3-ITD AML undergoing allo-HSCT might benefit from maintenance with FLT3 inhibitors post-transplantation [7–9, 23].

Sorafenib, a multi-kinase inhibitor, has shown promising efficacy in the treatment of FLT3-ITD AML, including maintenance post-transplantation [23–33]. Recently, two back-to-back RCTs including SORMAIN and our own have demonstrated that sorafenib maintenance can prevent relapse and improve survival for patients with FLT3-ITD AML following allo-​HSCT [35, 36]. SORMAIN, which was terminated early because of slow accrual, demonstrated that sorafenib maintenance could reduce the risk of relapse and death after allo-HSCT for patients with FLT3-ITD AML, with the 2-year overall survival (OS) of 90.5% and 66.2% in the sorafenib and placebo groups [36]. Our phase III RCT showed that sorafenib maintenance revealed a significant advantage over non-maintenance in relapse and OS, with the 2-year relapse rate and OS of 11.9% and 82.1% in the sorafenib group compared with 31.6% and 68.0% in the non-maintenance group [35]. Apart from the direct antileukemic effect of sorafenib, several studies including our own suggested the synergism between sorafenib and alloreactive donor T cells in promoting graft-versus-leukemia activity [35, 37, 38]. Therefore, the researchers of SORMAIN and we both proposed the prospective trials of sorafenib maintenance after allo­HSCT in AML patients without FLT3­ITD mutations [39].

Some retrospective and single-arm exploratory studies revealed that post-transplantation maintenance of other FLT3 inhibitors could also reduce relapse and improve survival for patients with FLT3-ITD AML [40, 41]. The preliminary result of a phase II RCT about midostaurin maintenance post-transplantation revealed that midostaurin maintenance could reduce the risk of relapse, with the 18-month leukemia-free survival (LFS) of 76% and 89% in the non-maintenance and midostaurin arms, respectively [42]. An ongoing phase III RCT is evaluating gilteritinib maintenance following allo-HSCT in patients with FLT3-ITD AML (NCT02997202) [43]. Further research is needed to ascertain which FLT3 inhibitors are most effective for post-transplantation maintenance in patients with FLT3-ITD AML. Key studies of FLT3 inhibitors as maintenance strategy in AML patients after allo-HSCT are summarized in Table 2.

**Table 2:** Key studies of FLT3 inhibitors as maintenance therapy in AML patients after allo-HSCT

References	Study design	Patients, N	Flt3 inhibitors, maintenance duration	Relapse	Survival
Chen [29]	Phase I	22 FLT3-ITD AML	Sorafenib, 12 months	3/22 (13.6%)	1-year OS: 95%; 1-year LFS: 85%
Brunner [30]	Retrospective, 2 arms	81 FLT3-ITD AML (Sorafenib: 26; Control: 55)	Sorafenib, median duration of 336.5 (19–1556) days	2-year CIR: 8.2% (Sorafenib) versus 37.7% (Control)	Sorafenib versus Control 2-year OS: 81% versus 62%; 2-year LFS: 82% versus 53%
Battipaglia [31]	Retrospective, single arm	27 FLT3-mutated AML (25 FLT3- ITD, 2 FLT3-TKD)	Sorafenib, median duration of 8.4 (0.2–46) months	3/27 (11.1%)	2-year OS: 80% ± 8% 2-year LFS: 73% ± 9%
Xuan [25]	Retrospective, 2 arms	144 FLT3-ITD AML (Sorafenib: 58; Control: 86)	Sorafenib, median duration of 146 (51–240) days	3-year CIR: 17.3% (Sorafenib) versus 34.2% (Control)	Sorafenib versus Control 3-year OS: 81.3% versus 62.9%; 3-year LFS: 79.3% versus 52.1%
Bazarbachi [33]	Retrospective, EBMT registry-based analysis	462 FLT3-mutated AML (Sorafenib: 28; Control: 434)	Sorafenib, median duration of ≥ 12 months	2-year CIR of total 462 patients: 34%	Matched-pair analysis 26 sorafenib patients versus 26 controls: 2-year OS: 83% versus 62%; 2-year LFS: 79% versus 54%
Xuan [35]	Phase III RCT	202 FLT3-ITD AML (Sorafenib: 100; Control: 102)	Sorafenib was administered at 30–60-day post-transplantation and continued until day 180	Sorafenib versus Control 1-year CIR: 7.0% versus 24.5%; 2-year CIR: 11.9% versus 31.6%	Sorafenib versus Control 2-year OS: 82.1% versus 68.0%; 2-year LFS: 78.9% versus 56.6%
Burchert [36]	Phase II RCT (SORMAIN, terminated early due to slow accrual)	83 FLT3-ITD AML (Sorafenib: 43; Placebo: 40)	Sorafenib, 24 months	Sorafenib (8/43, 18.6%) versus Placebo (17/40, 42.5%)	Sorafenib versus Placebo 2-year OS: 90.5% versus 66.2%; 2-year LFS: 85.0% versus53.3%
Sandmaier [40]	Phase I	13 AML	Quizartinib (AC220), a maximum of 24 months	1/13 (7.7%)	NA
Maziarz [42]	Phase II RCT (the preliminary result)	60 FLT3-ITD AML (Midostaurin + SOC: 30; SOC: 30)	Midostaurin, 12 months	18-month estimated relapse rate: 11% (Midostaurin + SOC) versus 24% (SOC)	18-month LFS: 89% (Midostaurin + SOC) versus 76% (SOC)
Levis [43]	Phase III RCT (Ongoing, NCT02997202)	346 FLT3-ITD AML (target number, Gilteritinib: 173; Placebo: 173)	Gilteritinib, 2 years	NA	NA

*Ref* reference, *N* number, *FLT3-ITD* FLT3 internal tandem duplication, *OS* overall survival, *LFS* leukemia-free survival, *CIR* cumulative incidence of relapse, *FLT3-TKD* FLT3 tyrosine kinase domain, *EBMT* European Society for Blood and Marrow Transplantation, *RCT* randomized controlled trial, *NA* not available, *SOC *standard of care

## Isocitrate dehydrogenase (IDH) inhibitors

Approximately 20% of AML genomes harbor mutations in one of two isoforms of IDH (IDH1 or IDH2). IDH mutation reduces α-ketoglutarate to 2-hydroxyglutarate, leading to histone hypermethylation and a block in myeloid differentiation [44]. AML patients with IDH mutation have a poor response to traditional chemotherapy and a higher relapse rate. Recently, ivosidenib and enasidenib, oral inhibitors of mutant IDH1 and IDH2, have shown good clinical response in relapsed/refractory or newly diagnosed IDH-mutated AML patients [45–47]. A phase I ongoing trial is evaluating the safety of enasidenib maintenance for IDH2-mutated myeloid neoplasms following allo-HSCT (NCT03515512).

## Venetoclax

Venetoclax, a selective small-molecular inhibitor of B-cell lymphoma-2 (Bcl-2), has been demonstrated that single-agent or combined with other agents are effective and tolerable to AML patients including relapsed or refractory AML patients, especially in elderly patients unfit for intensive chemotherapy, with overall response rate of 19–72% [48–50]. A phase I study demonstrated the feasibility of venetoclax combined with chemotherapy followed by venetoclax maintenance in fit elderly AML patients, with the median OS of 11.2 months [49]. Recently, Kent et al. reported that venetoclax was safe and tolerable as post-transplant maintenance for AML patients at high risk of relapse, with the 6-month LFS of 87% [51]. Currently, two trials are under way to evaluate venetoclax combined with azacitidine as maintenance for AML patients undergoing allo-HSCT (NCT04161885, NCT04128501).

## Histone deacetylase and hedgehog inhibitors

Histone deacetylase inhibitors (HDACi) are epigenetic modifiers that are shown to induce cell-cycle arrest, cell differentiation and apoptosis of AML cells. Panobinostat is an oral HDACi that has been reported to have moderate antileukemia activity in advanced AML and MDS patients [52]. A phase I/II study showed that panobinostat maintenance post-transplantation revealed favorable survival outcomes for high-risk MDS/AML patients compared with reports from similar patient groups [53].

The Hedgehog signaling pathway plays a critical role in embryonic development and aberrant Hedgehog signaling are deemed to contribute to the survival and expansion of leukemia stem cells [54]. Glasdegib, a Hedgehog pathway inhibitor, combined with low-dose cytarabine showed a favorable benefit-risk profile for AML patients unsuitable for intensive chemotherapy [55, 56]. A phase II study about glasdegib as maintenance for high-risk AML patients undergoing allo-HSCT is completed (NCT01841333), and the results have not been released.

## Maintenance duration

To date, the duration of maintenance of targeted drugs post-transplantation is not firmly established. In some retrospective and prospective studies, targeted drugs are usually maintained for 1–2 years after allo-HSCT [7, 8, 23]. Currently, there is a lack of RCTs on different maintenance duration of targeted drugs post-transplantation. In our phase III RCT, sorafenib was administered at 30–60-day post-transplantation and continued until day 180, with the 2-year LFS and OS of 78.9% and 82.1% [35]. Sorafenib was administered for 24 months in SORMAIN, with the 2-year LFS and OS of 85.0% and 90.5% [36]. Although our duration of sorafenib maintenance was favorable in terms of tolerance, patient’s quality of life and cost-effectiveness, prolonging sorafenib maintenance for 1 year or more post-transplantation might yield greater benefits for these patients. Besides, receptor- and nonreceptor-related mutations, epigenetic changes and signaling pathway alterations might contribute to resistance to FLT3 inhibitors [57]. Whether long-term exposure to sorafenib induces secondary gene mutations and drug resistance remains unclear. Our results showed that only one patient with sorafenib maintenance acquired an FLT3 tyrosine kinase domain mutation at relapse, and the CR rate and OS after salvage therapy were similar between two groups, suggesting that sorafenib maintenance for six-month post-transplantation did not increase the risk of mutation and resistance [35].

## Immunotherapies

Traditional immunotherapies such as donor lymphocyte infusion, interleukin-2 and interferon-α might play a role in preventing relapse for AML patients after allo-HSCT [11, 58]. However, their timing, dosing and co-administration with other agents require further study. Novel immunotherapeutic strategies including chimeric antigen receptor (CAR) T-cell therapy, antibody-directed therapy, immune checkpoint inhibitors and vaccines are under investigation [8, 59]. CAR T-cell therapy has yielded unprecedented efficacy in B cell malignancies, but there is limited data on its use in AML [59]. One of the major challenges in adopting CAR T-cell therapy in AML is the lack of an AML-specific antigen. Multiple targets for directed CAR T-cell therapy in AML include CD33, CD123, folate receptor β, NKG2D and Lewis Y [59]. Monoclonal antibodies such as anti-CD33, anti-CD123 and anti-CD45, bispecific antibodies and immune checkpoint inhibitors targeting programmed cell death protein-1 (PD-1) and cytotoxic T lymphocyte-associated antigen-4 (CTLA-4) are currently in early clinical trials [59]. The main categories of vaccines currently tested in AML are peptide vaccines and dendritic cell-based vaccines [59]. Wilms tumor-1 (WT-1) peptide vaccine was reported to be safe and potential as maintenance therapy in AML patients after allo-HSCT [60].

## Conclusion

Over the past decade, some novel targeted drugs have been explored for post-transplant maintenance for AML patients, and have shown promising results. Two back-to-back RCTs both reveal that sorafenib maintenance after allo­HSCT prevents relapse in patients with FLT3­ITD AML, resulting in an OS benefit. However, most of the studies about maintenance therapy in AML patients after allo-HSCT are retrospective and non-randomized. Currently, there is no consensus on the optimal duration of targeted drugs to prevent relapse, and it remains unclear whether long-term exposure to targeted drugs induces drug resistance. And which specific subpopulations of AML patients will benefit post-transplant maintenance most remains to be further investigation. Therefore, well-designed, fully powered, prospective trials are needed to address the unsettled questions to further improve the outcomes post-transplantation. Except for targeted drugs, some immunotherapies including CAR T-cell therapy, antibody-directed therapy, immune checkpoint inhibitors and vaccines have also attracted board attention.

## Data Availability

Not applicable.

## Abbreviations

AML: Acute myeloid leukemia
CR: Complete remission
allo-HSCT: Allogeneic hematopoietic stem cell transplantation
MRD: Measurable residual disease
MDS: Myelodysplastic syndrome
RCT: Randomized controlled trial
FLT3-ITD: FLT3 internal tandem duplication
OS: Overall survival
LFS: Leukemia-free survival
IDH: Isocitrate dehydrogenase
Bcl-2: B-cell lymphoma-2
HDACi: Histone deacetylase inhibitor
CAR: Chimeric antigen receptor
PD-1: Programmed cell death protein-1
CTLA-4: Cytotoxic T lymphocyte-associated antigen-4
WT-1: Wilms tumor-1
